# Comparable Fusion Response, but Increased Inflammatory Response, with *Escherichia coli*-Derived Recombinant Human Bone Morphogenetic Protein-2 in Posterior Lumbar Interbody Fusion Surgery

**DOI:** 10.3390/jcm15114026

**Published:** 2026-05-22

**Authors:** Mu Ha Lee, Hyun Jun Jang, Kyung Hyun Kim, Jeong-Yoon Park, Sung Uk Kuh, Dong Kyu Chin, Keun Su Kim, Jae Keun Oh, Bong Ju Moon

**Affiliations:** 1 Department of Neurosurgery, Soonchunhyang University Seoul Hospital, Seoul 04401, Republic of Korea; genus0614@gmail.com; 2 Department of Neurosurgery, Spine and Spinal Cord Institute, Gangnam Severance Hospital, Yonsei University College of Medicine, Seoul 06273, Republic of Korea; janghj0@gmail.com (H.J.J.); nskhk@yuhs.ac (K.H.K.); spinepjy@gmail.com (J.-Y.P.); kuhsu@yuhs.ac (S.U.K.); dkchin@yuhs.ac (D.K.C.); spinekks@yuhs.ac (K.S.K.); 3 Department of Neurosurgery, Kangbuk Samsung Hospital, Sungkyunkwan University School of Medicine, Seoul 03181, Republic of Korea

**Keywords:** spinal fusion, osteolysis, sclerosis, bone morphogenetic protein 2, *Escherichia coli*, lumbar vertebrae, inflammation

## Abstract

**Background/Objectives**: This retrospective study aimed to evaluate the radiologic outcomes and changes in biochemical inflammatory markers following posterior lumbar interbody fusion (PLIF) with *Escherichia coli*-derived recombinant human bone morphogenetic protein-2 (E.BMP-2), compared with conventional autologous bone grafting. **Methods**: The study included 112 patients undergoing single- or two-level PLIF for degenerative lumbar disease between 2022 and 2023, divided into E.BMP-2 (*n* = 50) and Control (*n* = 62) groups. Radiological outcomes, including Bridwell grading system and adjacent vertebral body (VB) changes, and changes in biochemical inflammatory markers—white blood cell (WBC) count, C-reactive protein (CRP), erythrocyte sedimentation rate (ESR), and neutrophil count—were assessed. Clinical outcomes were also evaluated. Multivariate regression and propensity-score-matched analyses, and linear mixed-effects models were applied. **Results**: Fusion rates were comparable between the groups (90.8% vs. 96.7%; *p* = 0.466); adjusted analyses showed no independent association between E.BMP-2 use and fusion outcomes. The E.BMP-2 group demonstrated a higher prevalence of adjacent VB changes (78.5% vs. 54.3%; *p* = 0.001), and higher postoperative inflammatory markers including CRP levels on postoperative day 7 and at 1 month, along with increased neutrophil levels on postoperative day 4 (CRP day 7: 31.7 ± 26.4 mg/L vs. 18.7 ± 14.4 mg/L, *p* = 0.014; CRP 1 month: 7.2 ± 13.0 mg/L vs. 2.7 ± 3.8 mg/L, *p* = 0.022; neutrophil count day 4: 64.4 ± 10.6% vs. 60.6 ± 8.7%, *p* = 0.039). However, no significant differences in clinical outcomes, as assessed by VAS scores, were observed according to adjacent VB changes or inflammatory markers. Postoperative fever and infection rates were similar between groups. **Conclusions**: E.BMP-2 use in PLIF demonstrated fusion rates comparable to those of autografts, without demonstrated superiority. No significant differences in clinical outcomes were identified. Further large-scale prospective studies are needed to clarify its clinical role and optimal dosing.

## 1. Introduction

As the population ages, the number of patients undergoing surgery for degenerative spinal diseases is continuing to rise [1]. Hence, fusion surgery for lumbar spinal diseases is widely performed [2]. The types of fusion surgeries include posterolateral fusion (PLF), anterior lumbar interbody fusion (ALIF), oblique lumbar interbody fusion (OLIF), lateral lumbar interbody fusion (LLIF), posterior lumbar interbody fusion (PLIF), and transforaminal lumbar interbody fusion (TLIF). Among these, posterior approaches such as PLIF and TLIF are more frequently performed because they can extensively remove pain generators, result in relatively lower complication rates, and are associated with good prognosis [3,4].

Bone grafting is commonly performed inside a cage to promote interbody fusion, and a broad selection of graft materials may be used. While autologous bone grafts have traditionally been favored for fusion, technological advances have facilitated the widespread use of allografts, demineralized bone matrix (DBM), ceramics, recombinant human bone morphogenetic protein-2 (rh-BMP-2), and anorganic bone matrix/15-amino acid peptide fragments (ABM/P-15) [5,6].

Among these graft options, rh-BMP-2 has demonstrated high efficacy in ALIF. In 2002, rh-BMP-2, marketed as Infuse (Medtronic, Minneapolis, MN), a Chinese hamster ovary (CHO)-derived rh-BMP-2 (C.BMP-2), was approved by the US Food and Drug Administration (FDA) for use in single-level ALIF [7]. However, its use in PLIF is off-label and concerns regarding safety and efficacy persist. Recently, a novel form of rh-BMP-2, *Escherichia coli*-derived rh-BMP-2 (E.BMP-2), has been developed. Unlike C.BMP-2, there are few studies on E.BMP-2, and most available studies have focused on PLF. Investigations specifically addressing PLIF are rare. Furthermore, most previous studies have primarily focused on fusion rates and clinical outcomes. Only a limited number of studies have systematically evaluated postoperative changes in biochemical markers or radiologic findings after PLIF [8,9,10]. Rh-BMP-2 is known to trigger a transient inflammatory response which may be reflected in postoperative biochemical markers. Radiologic changes such as osteolysis or adjacent vertebral body alterations can occur following its use [10,11]. However, the relationship between early postoperative biochemical changes and later radiologic changes remains unclear, particularly in the context of PLIF using E.BMP-2. Therefore, in this study, we aimed to investigate postoperative changes in biochemical markers and radiological findings following the use of E.BMP-2 in PLIF, and to explore their potential association with clinical outcomes.

## 2. Materials and Methods

### 2.1. Patients and Study Design

**Figure 1:**
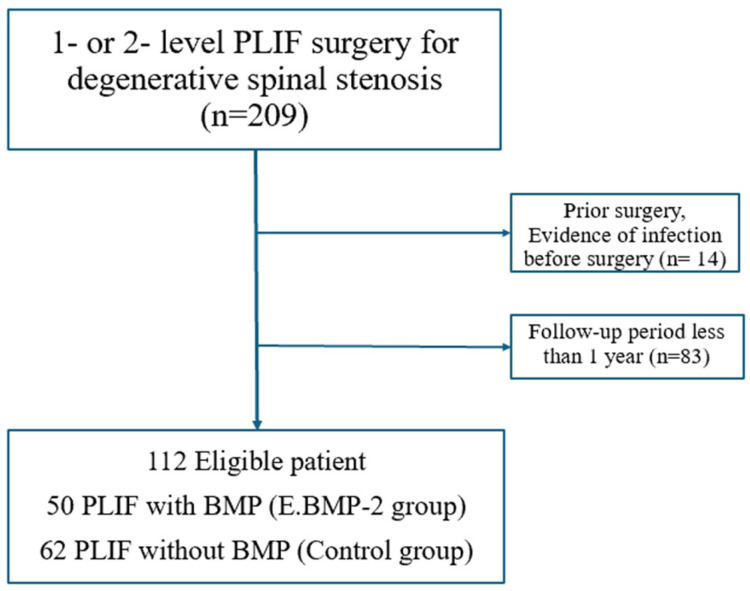
Patient selection algorithm. BMP, bone morphogenetic protein; E.BMP-2, *Escherichia coli*-derived recombinant human bone morphogenetic protein-2; PLIF, posterior lumbar interbody fusion.

This retrospective study was conducted at a single institution from 2022 to 2023. The study was approved by the Institutional Review Board (IRB) of Yonsei University College of Medicine, Gangnam Severance Hospital (IRB No.3-2024-0432). The inclusion criteria included patients who underwent PLIF with local autologous bone or E.BMP-2/hydroxyapatite (HA) for degenerative lumbar disease with persistent symptoms that were unresponsive to adequate conservative management. Eligible patients underwent either single- or two-level PLIF. The exclusion criteria included a history of spinal surgery; history of surgery for trauma, infection, tumors, or conditions unrelated to degenerative lumbar disease; those with preoperative evidence of other infections; and patients with a follow-up duration of less than 1 year (Figure 1).

### 2.2. Surgical Methods

All surgeries were performed under general anesthesia, and a standard posterior approach was used in all patients. The patients were placed in a prone position on a Jackson table. A midline skin incision measuring 10–12 cm was made, followed by subperiosteal dissection. Following exposure of the lamina and mammillary processes, subtotal laminectomy and extensive facetectomy were performed to expose the intervertebral disc. The nucleus and cartilaginous end plates were removed, and bilateral annulotomy was performed. Disc shavers and curettes were used to prepare the endplates, with care taken to avoid damage to the bony endplates. PEEK cages (Lumfix cage, CGBio Co., Ltd., Seoul, Republic of Korea) were used for both the E.BMP-2 and Control groups. In the E.BMP-2 group, the cages were filled with autologous bone from the laminectomy site in combination with E.BMP-2 and HA granules (Novosis; CG Bio Co., Ltd., Seoul, Republic of Korea), whereas the cages in the Control group were filled with autologous bone from the laminectomy site. An amount of 0.5 mg E.BMP-2 was used at each level based on a previous study [10]. After endplate preparation, the autologous bone and DBM were packed into the disc space, and the cage was inserted tightly into the disc space using a root retractor while protecting the nerve. Gelfoam and fibrin glue were applied to seal the annulotomy sites and minimize E.BMP-2 leakage. Pedicle screw fixation was performed after cage insertion. After confirming hemostasis, the wound was closed layer-by-layer.

### 2.3. Outcome Measures

Demographic and perioperative data such as sex, age, length of hospital stay, number of operated levels, body mass index (BMI), presence of osteoporosis, comorbidities (hypertension, diabetes, cardiovascular disease, pulmonary disease, and kidney disease), alcohol consumption, smoking status, and complications such as postoperative fever and infection were collected and analyzed. Clinical outcomes were assessed using the visual analog scale (VAS) preoperatively, 1 month postoperatively and at 1 year postoperatively.

Biochemical inflammatory markers, including white blood cell (WBC) count, erythrocyte sedimentation rate (ESR), C-reactive protein (CRP), and neutrophil levels, were also assessed. Blood serum samples were obtained the day before surgery, with additional postoperative samples collected on day 4, day 7, and at 1 month.

**Figure 2:**
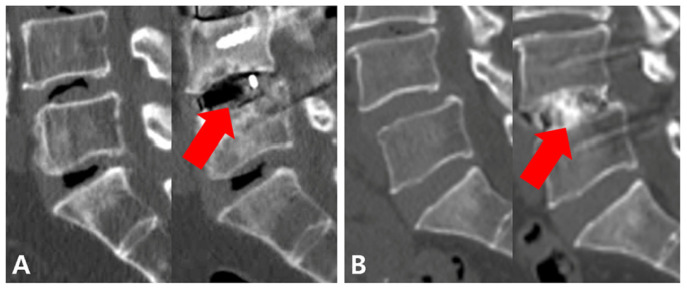
Fusion assessed with the Bridwell interbody fusion grading system: (**A**) is a patient with cages filled with *Escherichia coli*-derived recombinant human bone morphogenetic protein-2 and autologous bone (E.BMP-2 group); (**B**) is a patient with cages filled with autologous bone only (Control group). Fusion was graded from I to IV: fused with remodeling and trabeculae present (Grade I); graft was intact, not fully remodeled and incorporated, but no lucency present (Grade II); graft was intact, potential lucency present at top and bottom of graft (Grade III); and fusion was absent with collapse/resorption of graft (Grade IV). According to the Bridwell grading system, fusion is defined as Grade I and II based on radiologic outcomes. (**A**) is Grade IV and (**B**) is Grade I fusion. Red arrows indicate the interbody fusion status and representative graft appearance at the operated level.

**Figure 3:**
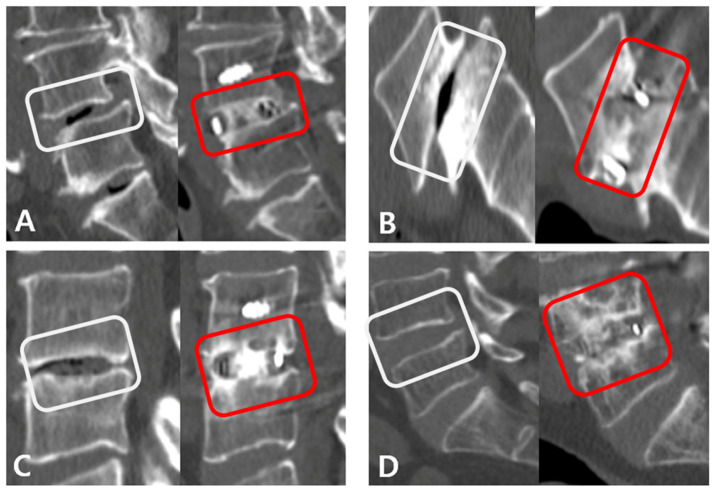
Adjacent vertebral body changes. We classified adjacent vertebral body changes, which are a comparison of preoperative computed tomography scan and at 1 year follow-up, into 4 groups: no change (**A**), osteolysis (**B**), sclerosis (**C**), and sclerosis with osteolysis (**D**). The white box represents the preoperative adjacent vertebral body, and the red box represents the corresponding region at the 1-year follow-up.

Radiological outcomes were evaluated using computed tomography (CT). CT was performed preoperatively and at 1 year postoperatively. The bone fusion status was assessed according to the Bridwell interbody fusion grading system based on CT [12]. The Bridwell fusion grading system was defined as follows: Grade I, fused with remodeling and trabeculae present; Grade II, graft was intact, not fully remodeled and incorporated, but no lucency was present; Grade III, graft was intact, potential lucency was present at the top and bottom of the graft; and Grade IV, fusion was absent with collapse/resorption of the graft (Figure 2). Adjacent vertebral body changes were determined by comparing preoperative CT scans and 1-year follow-up and classified into four categories: no change, osteolysis, sclerosis, and sclerosis combined with osteolysis (Figure 3). Radiologic outcomes, including fusion status based on Bridwell grading system, were assessed by two independent spine surgeons. In case of disagreement, a consensus was reached through joint review and discussion. The complications were also analyzed.

### 2.4. Statistical Analysis

All statistical analyses were performed using R statistical software version 4.2.3 (R Foundation for Statistical Computing, Vienna, Austria). Continuous variables were presented as mean ± standard deviation, depending on the data distribution, and categorical variables were expressed as counts and percentages. The normality of continuous variables was assessed using the Shapiro–Wilk test. For normally distributed variables, between-group comparisons were performed using the independent t-test or one-way analysis of variance (ANOVA). For non-normally distributed variables, non-parametric tests (Mann–Whitney U test or Kruskal–Wallis test) were applied. Categorical variables were compared using the chi-square test or Fisher’s exact test, as appropriate. Correlation analyses were performed using Pearson’s correlation to explore potential associations between variables. To adjust for potential confounding, multivariate regression analysis was performed including age, sex, and the number of operated levels. In addition, propensity score matching was conducted using these variables. To account for repeated measurements over time, linear mixed-effects models were applied, including fixed effects for group, time, and group–time interaction with subject-level random effects. Effect sizes were reported as odds ratios for categorical outcomes, along with corresponding 95% confidence intervals (CIs). Statistical significance was defined as a *p*-value of less than 0.05.

## 3. Results

### 3.1. Patient Characteristics

A total of 112 patients were enrolled in this study (E.BMP-2 group, *n* = 50; Control group, *n* = 62). The proportion of females was 54% in the E.BMP-2 group and 79.0% in the Control group. The mean age in the E.BMP-2 group was 66.3 ± 8.8 years, whereas in the Control group it was 69.3 ± 6.1 years. The average operation level was 1.3 ± 0.5 for the E.BMP-2 group and 1.5 ± 0.5 for the Control group. No significant differences were observed between the two groups regarding length of hospital stay, BMI, presence of osteoporosis, comorbidities (hypertension, diabetes mellitus, cardiovascular disease, respiratory disease, and renal disease), alcohol consumption, and smoking status. Similarly, there were no significant differences in complications, including postoperative fever and infection, or in clinical outcomes (Table 1).

### 3.2. Radiologic Outcomes

When comparing radiologic outcomes per segment, no significant difference was observed between the groups regarding the Bridwell fusion grade at the 1-year CT assessment. Regarding adjacent vertebral body (VB) changes, a significant difference was observed between the groups. The E.BMP-2 group demonstrated a higher rate of adjacent VB changes (78.5% vs. 54.3%), corresponding to an absolute risk difference of 24.2% (95% CI, 10.0% to 38.4%; *p* = 0.001). No significant differences were observed in radiologic complications between the two groups (Table 2).

### 3.3. Biochemical Inflammatory Markers and Clinical Outcomes

Both groups experienced peak WBC, CRP, and neutrophil levels on day 4, followed by a subsequent decline. The ESR peaked on day 7 and decreased thereafter. Inter-group comparisons revealed that CRP levels on day 7 and at 1 month differed significantly, with the E.BMP-2 group showing CRP values of 31.7 ± 26.4 mg/L and 7.2 ± 13.0 mg/L, and the Control group showing values of 18.7 ± 14.4 mg/L and 2.7 ± 3.8 mg/L, respectively (day 7: *p* = 0.014, 1 month: *p* = 0.022). Neutrophil levels on postoperative day 4 were also higher in the E.BMP-2 group (64.4 ± 10.6% vs. 60.6 ± 8.7%; *p* = 0.039). No significant differences in WBC count or ESR were observed between the groups (Table 3). Linear mixed-effects modeling demonstrated no significant group–time interaction for WBC, ESR, or neutrophil levels, indicating comparable temporal patterns across groups. In contrast, a significant group–time interaction was observed for CRP level (*p* = 0.004) (Supplementary Table S1).

**Figure 4:**
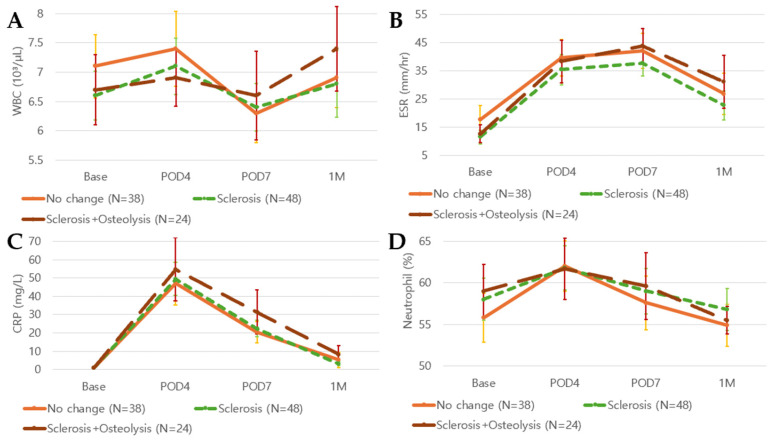
Biochemical inflammatory marker trends over time according to adjacent vertebral body (VB) changes. We analyzed biochemical inflammatory markers according to VB changes (no change, *n* = 38; sclerosis, *n* = 48; sclerosis + osteolysis, *n* = 24). WBC levels (10^3^/μL) over time (**A**), ESR levels (mm/h) over time (**B**), CRP levels (mg/L) over time (**C**), and neutrophil levels (%) over time (**D**). CRP, C-reactive protein; ESR, erythrocyte sedimentation rate; POD, postoperative day; WBC, white blood cell. Data are presented as mean values with 95% confidence interval. Note: Due to the limited sample size for the osteolysis group, this group was omitted from subsequent analyses between groups.

**Figure 5:**
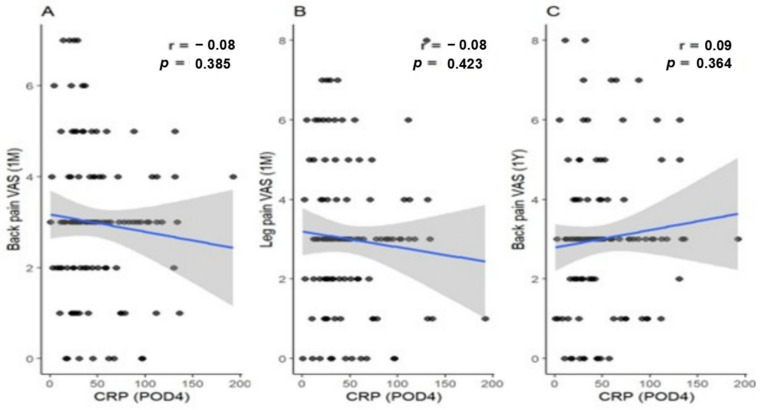
Association between early postoperative CRP levels and pain outcomes. Scatter plots showing the relationship between CRP levels at postoperative day 4 and pain scores (VAS): (**A**) back pain at 1 month, (**B**) leg pain at 1 month, and (**C**) back pain at 1 year. Each dot represents an individual patient. Solid lines indicate linear regression with 95% confidence intervals.

When analyzed according to adjacent VB changes (no change, *n* = 38; osteolysis, *n* = 2; sclerosis, *n* = 48; sclerosis with osteolysis, *n* = 24), similar temporal patterns were observed across most groups. WBC, CRP, and neutrophil counts peaked on postoperative day 4 and consistently declined, while ESR peaked on day 7 and subsequently decreased (Supplementary Table S2, Supplementary Figure S1). Among the remaining groups, no significant differences were observed in postoperative CRP levels or VAS scores (Table 4 and Figure 4). Correlation analyses demonstrated no significant association between early postoperative CRP levels (postoperative day 4) and VAS scores (all *p* > 0.05) (Figure 5). In addition, Pearson correlation analyses showed no significant correlation between VAS score and inflammatory markers (all |r| < 0.3) (Supplementary Figure S2).

### 3.4. Clinical Characteristics According to Fusion Status

In a per-level analysis comparing the fusion group (*n* = 103) and the non-fusion group (*n* = 9), the non-fusion group had a significantly greater proportion of two-level surgeries (77.8% vs. 36.9%, *p* = 0.041). At the 1-year follow-up, the non-fusion group also showed a significantly higher incidence of sclerosis with osteolysis compared with the fusion group (55.6% vs. 18.4%, *p* = 0.032). No significant differences were observed between groups in terms of sex, age, BMI, E.BMP-2 use, or osteoporosis status. No significant differences were observed in VAS back scores at 1 year postoperatively (4.3 ± 2.7 vs. 2.9 ± 1.7, *p* = 0.161). In contrast, VAS leg scores at 1 year were significantly lower in the fusion group than in the non-fusion group (2.8 ± 2.0 vs. 4.8 ± 2.0, *p* < 0.005) (Table 5).

### 3.5. Results of Propensity-Score-Matched Analysis

In the multivariate logistic regression analysis, sex and the number of operated levels were identified as significant factors associated with fusion, whereas E.BMP-2 was not significantly associated with fusion outcomes. Consistently, E.BMP-2 was also not found to be significantly associated with fusion in the propensity-score-matched analysis adjusting for age, sex, and the number of operated levels (odds ratio, 0.47; 95% CI, 0.09–1.89; *p* = 0.303) (Table 6).

## 4. Discussion

As society ages, the prevalence of degenerative spinal diseases continues to increase [1,2]. Non-union remains one of the most common complications of lumbar fusion and may lead to pain, instability, and poor clinical outcomes. Reported non-union rates vary widely, and several risk factors, including age, smoking, and the number of fused levels, have been related to fusion failure [13,14]. To enhance fusion outcomes, various bone graft materials and osteoinductive agents including rh-BMP-2, have been introduced and widely investigated in spinal fusion surgery [5,6].

Recombinant human BMP-2 (rh-BMP-2) is an osteoinductive agent widely used in spinal fusion. While its use is approved for ALIF, applications in PLIF remain off-label. Recently, E.BMP-2 has been developed as an alternative to C.BMP-2, with comparable osteoinductive properties reported in experimental and clinical studies [15,16]. However, few studies have investigated the use of E.BMP-2 in PLIF surgery, and most available studies have focused on PLF surgery [8,9,10,17]. Therefore, we conducted this study to evaluate the efficacy of E.BMP-2 in PLIF. A previous study demonstrated that 0.5 mg of E.BMP-2 represents the minimum effective dose required to achieve fusion in PLF surgery [8], and that 0.3–0.5 mg per level may be sufficient in PLIF surgery [10]. In this study, the E.BMP-2 group received 0.5 mg of E.BMP-2 per level in combination with autologous bone and DBM, whereas the Control group received autologous bone and DBM. In the present study, no significant difference in fusion rate was observed between groups, and E.BMP-2 was not significantly associated with fusion outcomes in adjusted analyses. These findings suggest that E.BMP-2 was not associated with improved fusion outcomes at the applied dose. Although differences in postoperative inflammatory responses and bone remodeling patterns have been reported in previous studies [18], any such interpretations in this study remain speculative, as it was not designed to investigate underlying biological mechanisms. In addition, although several baseline variables, including BMI, osteoporosis, and comorbidities, were comparable between groups, they were not included in the adjusted models due to the limited sample size and concerns regarding model overfitting. The inclusion of these variables in sensitivity analyses may have further strengthened confounding control. Therefore, residual confounding cannot be excluded. Furthermore, as no direct comparisons across different E.BMP-2 dosage levels were performed, conclusions regarding optimal dosing cannot be drawn. Therefore, further studies are required to clarify the optimal and minimum effective dose of E.BMP-2 in PLIF surgery.

Complications associated with the use of BMP in lumbar fusion surgery include vertebral osteolysis, graft subsidence, postoperative radiculitis, postoperative seroma or hematoma, ectopic bone formation, and retrograde ejaculation [19,20,21,22]. Although higher doses of BMP may enhance fusion rates, they may also increase the risk of adverse events [23]; therefore, identifying the minimum effective dose remains important.

Few studies have investigated changes in biochemical markers and radiological findings following PLIF surgery. In uncomplicated postoperative courses, inflammatory markers typically show transient elevations, whereas persistent or secondary increases may suggest infection [24,25,26,27]. In our study, both groups showed similar temporal patterns of postoperative inflammatory markers, with early postoperative peaks followed by gradual decline, while ESR peaked later. Although CRP showed a significant group-time interaction, these differences were not consistently associated with clinical outcomes or radiologic changes. Therefore, a direct causal or clinically predictive relationship between early postoperative CRP levels and long-term structural changes cannot be established. With respect to radiologic findings, the E.BMP-2 group demonstrated a higher prevalence of both sclerosis and osteolysis in adjacent VB changes compared with the Control group. These findings are consistent with previously reported bone remodeling changes [28,29]; however, no significant associations were identified between these radiologic findings and systemic inflammatory markers, and the underlying biological mechanisms cannot be determined from the present data.

In addition, we assessed the relationship between VAS scores and both radiologic findings and biochemical inflammatory markers. No significant differences were observed in inflammatory markers or VAS scores across subgroups, and no significant correlations were identified between VAS scores and either inflammatory markers or radiologic parameters. Given the small sample size of the osteolysis subgroup and multiple comparisons performed without formal adjustment, these analyses should be considered hypothesis-generating, and their clinical relevance remains unclear.

This study has several limitations. First, the sample size was relatively small, particularly for subgroup analyses, which may have limited statistical power. Second, the 1-year follow-up period may be insufficient to fully evaluate bone fusion after PLIF surgery. Third, clinical outcomes were assessed using only VAS scores without validated functional outcome measures, such as the Oswestry Disability Index (ODI). Fourth, although multivariate regression and propensity-score-matched analyses were performed, residual confounding cannot be excluded. Furthermore, as this was an observational study, causal relationships cannot be established. Fifth, multiple comparisons were performed without formal adjustment, which may increase the risk of type I error. Finally, as a single-center retrospective study, the generalizability of these findings is limited. Further prospective studies with larger sample sizes and longer follow-up are required to validate these findings.

## 5. Conclusions

Following the use of E.BMP-2, fusion rates were comparable to those achieved with autologous bone grafts in PLIF surgery, without increased postoperative fever and infection. No significant differences were observed in clinical outcomes, including VAS scores, according to adjacent VB changes or inflammatory markers. Overall, E.BMP-2 did not demonstrate superiority over standard grafting techniques under the conditions of this study. While it may represent a viable alternative, further large-scale, prospective studies with a long follow-up and dose–response analyses are warranted.

## Figures and Tables

**Table 1:** Demographics and perioperative data.

	E.BMP-2 Group	Control Group	*p*-Value
	(N = 50)	(N = 62)	
Sex			**0** **.009**
Female	27 (54.0%)	49 (79.0%)	
Male	23 (46.0%)	13 (21.0%)	
Age (years)	66.3 ± 8.8	69.3 ± 6.1	**0** **.038**
Length of stay (days)	7.9 ± 1.9	8.5 ± 1.8	0.072
Level	1.3 ± 0.5	1.5 ± 0.5	**0** **.049**
BMI (kg/m^2^)	24.9 ± 3.1	24.9 ± 3.1	0.897
Osteoporosis	8 (16.0%)	16 (25.8%)	0.305
Hypertension	25 (50.0%)	36 (58.1%)	0.509
Diabetes	12 (24.0%)	20 (32.3%)	0.452
Cardiovascular disease	7 (14.0%)	15 (24.2%)	0.267
Pulmonary disease	11 (22.0%)	19 (30.6%)	0.417
Kidney disease	3 (6.0%)	3 (4.8%)	1.000
Alcohol	6 (12.0%)	6 (9.7%)	0.930
Smoking	4 (8.0%)	5 (8.1%)	1.000
Postoperative fever	3 (6.0%)	3 (4.8%)	1.000
Postoperative infection	0 (0.0%)	0 (0.0%)	1.000
VAS back			
Preoperative	6.6 ± 2.2	6.5 ± 1.4	0.849
1 Month	3.1 ± 1.5	2.9 ± 1.8	0.556
1 Year	3.2 ± 1.8	2.9 ± 1.9	0.344
VAS leg			
Preoperative	7.1 ± 1.7	6.6 ± 1.5	0.065
1 Month	3.0 ± 1.6	3.0 ± 2.1	0.841
1 Year	3.1 ± 2.1	2.7 ± 2.0	0.259

Descriptive data represents mean ± standard deviation. Bold indicates statistical significance. BMI, body mass index; E.BMP-2, *Escherichia coli*-derived recombinant human bone morphogenetic protein-2; VAS, visual analog scale.

**Table 2:** Radiologic outcomes (per level).

		E.BMP-2 Group	Control Group	*p*-Value
		(N = 65)	(N = 92)	
**Bridwell fusion grade (at 1-year follow-up)**				0.466
	Grade I	42 (64.6%)	62 (67.4%)	
	Grade II	17 (26.2%)	27 (29.3%)	
	Grade III	4 (6.2%)	2 (2.2%)	
	Grade IV	2 (3.1%)	1 (1.1%)	
**Adjacent VB change (at 1-year follow-up)**				**0** **.001**
	No change	14 (21.5%)	42 (45.7%)	
	Osteolysis	3 (4.6%)	0 (0.0%)	
	Sclerosis	27 (41.5%)	37 (40.2%)	
	Sclerosis + Osteolysis	21 (32.3%)	13 (14.1%)	
**Complication** *				1.000
	No	62 (95.4%)	88 (95.7%)	
	Yes	3 (4.6%)	4 (4.3%)	

Descriptive data represents mean ± standard deviation. Boldface indicates statistical significance. E.BMP-2, *Escherichia coli*-derived recombinant human bone morphogenetic protein-2; VB, vertebral body. * In E.BMP-2 group, one case of cage subsidence and screw fracture and two cases of cage retropulsion occurred. In control group, four cases of cage subsidence occurred.

**Table 3:** Biochemical inflammatory markers.

	E.BMP-2 Group	Control Group	*p*-Value
	(N = 50)	(N = 62)	
**WBC (10^3^/μL)**			
Preoperative	6.9 ± 1.7	6.7 ± 1.4	0.460
Day 4	7.5 ± 2.5	7.0 ± 1.2	0.155
Day 7	6.6 ± 1.8	6.3 ± 1.5	0.409
1 month	7.0 ± 1.7	7.0 ± 1.8	0.995
**ESR (mm/h)**			
Preoperative	11.7 ± 9.6	15.4 ± 12.9	0.086
Day 4	38.0 ± 19.5	36.6 ± 19.6	0.698
Day 7	42.6 ± 16.2	38.6 ± 18.3	0.304
1 month	25.7 ± 23.1	25.7 ± 19.4	0.997
**CRP (mg/L)**			
Preoperative	0.9 ± 0.8	0.9 ± 1.1	0.867
Day 4	52.6 ± 42.1	47.5 ± 30.9	0.477
Day 7	31.7 ± 26.4	18.7 ± 14.4	**0** **.014**
1 month	7.2 ± 13.0	2.7 ± 3.8	**0** **.022**
**Neutrophil (%)**			
Preoperative	57.5 ± 9.4	57.2 ± 8.5	0.870
Day 4	64.4 ± 10.6	60.6 ± 8.7	**0** **.039**
Day 7	60.1 ± 10.6	58.0 ± 9.4	0.333
1 month	55.5 ± 9.4	55.6 ± 7.3	0.954

Descriptive data represents mean ± standard deviation. Boldface indicates statistical significance. WBC, white blood cell; ESR, erythrocyte sedimentation rate; CRP, C-reactive protein; E.BMP-2, *Escherichia coli*-derived recombinant human bone morphogenetic protein-2.

**Table 4:** Biochemical inflammatory markers and clinical outcomes according to adjacent VB change except osteolysis group.

	No Change (NC)	Sclerosis (S)	Sclerosis + Osteolysis (SO)	*p* Value
	(N = 38)	(N = 48)	(N = 24)	
**WBC (10^3^/μL)**				
Preoperative	7.1 ± 1.7	6.6 ± 1.5	6.7 ± 1.5	0.371
Day 4	7.4 ± 2.0	7.1 ± 1.7	6.9 ± 1.2	0.507
Day 7	6.3 ± 1.6	6.4 ± 1.4	6.6 ± 1.9	0.787
1 month	6.9 ± 1.6	6.8 ± 2.0	7.4 ± 1.8	0.443
**ESR (mm/h)**				
Preoperative	17.6 ± 16.0	11.4 ± 8.4	12.6 ± 7.9	**0.043** *
Day 4	39.6 ± 20.3	35.4 ± 18.9	38.4 ± 19.0	0.588
Day 7	42.1 ± 19.7	37.7 ± 15.7	43.7 ± 15.8	0.391
1 month	26.8 ± 23.3	22.7 ± 18.2	31.1 ± 23.6	0.322
**CRP (mg/L)**				
Preoperative	1.0 ± 1.1	0.8 ± 1.1	0.8 ± 0.6	0.578
Day 4	46.9 ± 36.7	49.5 ± 31.6	54.7 ± 43.1	0.714
Day 7	20.4 ± 18.5	22.4 ± 15.6	31.4 ± 29.9	0.171
1 month	5.2 ± 13.2	3.3 ± 4.2	8.2 ± 11.9	0.178
**Neutrophils (%)**				
Preoperative	55.8 ± 9.3	58.0 ± 8.9	59.0 ± 8.0	0.350
Day 4	62.0 ± 9.5	61.8 ± 9.4	61.7 ± 9.2	0.989
Day 7	57.6 ± 10.2	59.0 ± 9.6	59.6 ± 10.1	0.745
1 month	54.9 ± 7.9	56.8 ± 8.8	55.5 ± 4.2	0.564
**VAS back**				
Preoperative	6.5 ± 1.6	6.4 ± 2.2	6.7 ± 1.5	0.806
1 Month	2.9 ±1.7	2.9 ± 1.4	3.4 ± 1.9	0.643
1 Year	3.0 ± 1.8	2.8 ± 1.7	3.6 ± 2.2	0.451
**VAS leg**				
Preoperative	6.9 ± 1.5	7.0 ± 1.8	6.7 ± 1.4	0.803
1 Month	3.1 ± 1.9	2.9 ± 1.5	3.3 ± 2.2	0.568
1 Year	3.0 ± 2.0	2.9 ± 1.9	3.0 ± 2.4	0.903

Descriptive data represents mean ± standard deviation. Boldface indicates statistical significance. VB, vertebral body; WBC, white blood cell; ESR, erythrocyte sedimentation rate; CRP, C-reactive protein; VAS, visual analog scale. * In post hoc test of ESR at preoperative, significant differences were found between the following groups: NC vs. S, *p* = 0.039; NC vs. SO, *p* = 0.229; S vs. SO, *p* = 0.901.

**Table 5:** Comparison between non-fusion and fusion groups.

	Non-Fusion Group *	Fusion Group **	*p*-Value
	(N = 9)	(N = 103)	
**Sex**			0.052
Female	3 (33.3%)	73 (70.9%)	
Male	6 (66.7%)	30 (29.1%)	
**Age (years)**	68.8 ± 6.55	67.9 ± 7.66	0.732
**Level**			**0** **.041**
1 level	2 (22.2%)	65 (63.1%)	
2 level	7 (77.8%)	38 (36.9%)	
**Adjacent VB change (at 1-year follow-up)**			**0** **.0** **32**
No change	0 (0.0%)	38 (36.9%)	
Osteolysis	0 (0.0%)	2 (1.9%)	
Sclerosis	4 (44.4%)	44 (42.7%)	
Sclerosis + Osteolysis	5 (55.6%)	19 (18.4%)	
**BMI (kg/m^2^)**	25.3 ± 3.5	24.9 ± 3.1	0.696
**E.BMP-2 Use**			0.300
No	3 (33.3%)	59 (57.3%)	
Yes	6 (66.7%)	44 (42.7%)	
**Osteoporosis**			1.000
No	7 (77.8%)	81 (78.6%)	
Yes	2 (22.2%)	22 (21.4%)	
**VAS back**			
Preoperative	6.0 ± 1.1	6.6 ± 1.9	0.369
1 Month	3.0 ± 1.3	3.0 ± 1.7	0.973
1 Year	4.3 ± 2.7	2.9 ± 1.7	0.161
**VAS leg**			
Preoperative	6.3 ± 0.9	6.9 ± 1.7	0.280
1 Month	3.6 ± 2.1	3.0 ± 1.8	0.343
1 Year	4.8 ± 2.0	2.8 ± 2.0	**0** **.005**

Descriptive data represents mean ± standard deviation. Boldface indicates statistical significance. VB, vertebral body; BMI, body mass index; E.BMP-2, *Escherichia coli*-derived recombinant human bone morphogenetic protein-2; VAS, visual analog scale. * Non-fusion group is Bridwell interbody fusion Grade III, IV. ** Fusion group is Bridwell interbody fusion Grade I, II.

**Table 6:** Association with fusion: multivariate and propensity-score-matched analysis.

		Odds Ratio	95% CI	*p*-Value
**Multivariate logistic regression**				
	E.BMP-2	0.34	0.06–1.79	0.215
	Age	0.97	0.85–1.11	0.684
	Sex (Male vs. Female)	0.14	0.02–0.68	**0.020**
	Operated level (2 vs. 1)	0.08	0.01–0.43	**0.007**
**Propensity-score-matched analysis** *				
	E.BMP-2	0.47	0.09–1.89	0.303

Boldface indicates statistical significance; E.BMP-2, *Escherichia coli*-derived recombinant human bone morphogenetic protein-2; CI, confidence interval. * Propensity score matching was performed using age, sex, and number of operated levels.

## Data Availability

The data presented in this study are available on request from the corresponding authors. Due to the presence of sensitive patient information, the dataset cannot be made publicly accessible to protect participant confidentiality and to comply with institutional and ethical regulations.

## Supplementary Materials

The following supporting information can be downloaded at: https://www.mdpi.com/article/10.3390/jcm15114026/s1, Figure S1: Biochemical inflammatory marker trends over time according to adjacent vertebral body (VB) changes.; Figure S2: Pearson correlation between inflammatory markers and pain scores; Table S1: Comparison of longitudinal changes in inflammatory markers between groups using linear mixed-effects model; Table S2: Biochemical inflammatory markers and clinical outcome according to adjacent VB change.

## Abbreviations

The following abbreviations are used in this manuscript:

PLIF	Posterior lumbar interbody fusion
E.BMP-2	*Escherichia coli*-derived recombinant human bone morphogenetic protein-2
VB	Vertebral body
WBC	White blood cell
CRP	C-reactive protein
ESR	Erythrocyte sedimentation rate
VAS	Visual analog scale
PLF	Posterolateral fusion
ALIF	Anterior lumbar interbody fusion
OLIF	Oblique lumbar interbody fusion
LLIF	Lateral lumbar interbody fusion
TLIF	Transforaminal lumbar interbody fusion
DBM	Demineralized bone matrix
rh-BMP-2	Recombinant human bone morphogenetic protein-2
ABM/P-15	Anorganic bone matrix/15-amino acid peptide fragments
C.BMP-2	Chinese hamster ovary-derived recombinant human bone morphogenetic protein-2
HA	Hydroxyapatite
BMI	Body mass index
CT	Computed tomography
POD	Postoperative day
RANKL	Receptor activator of nuclear factor kappa B ligand
