# Supplementary material for: Comparable Fusion Response, but Increased Inflammatory Response, with Escherichia coli-Derived Recombinant Human Bone Morphogenetic Protein-2 in Posterior Lumbar Interbody Fusion Surgery
Source: J Clin Med. 2026 May 22;15(11):4026. doi: 10.3390/jcm15114026 (PMC13257914; doi:10.3390/jcm15114026)
Supplement: Supplementary file 1 [file jcm-15-04026-s001.zip › jcm-4245478-supplementary.pdf]

Supplementary Table S1. Comparison of longitudinal changes in inflammatory markers between groups using linear mixed-effects model.

Markers	Group x Time p-value
WBC	0.432
ESR	0.238
Neutrophil	0.112
CRP (log-transformed)	**0.004**

Values represent p-values for the group-by-time interaction derived from linear mixed-effects models. Boldface indicates statistical significance. CRP were log-transformed to account for skewed distribution. WBC, white blood cell; ESR, erythrocyte sedimentation rate; CRP, c-reactive protein.

Supplementary Table S2. Biochemical inflammatory markers and clinical outcome according to adjacent VB change.

	No Change (NC) (N=38)	Osteolysis (O) (N=2)	Sclerosis (S) (N=48)	Sclerosis + Osteolysis (SO) (N=24)	p-Value
**WBC (10^3/μL)**					
Preoperative	7.1 ± 1.7	6.0 ± 0.7	6.6 ± 1.5	6.7 ± 1.5	0.477
Day 4	7.4 ± 2.0	12.2 ± 4.9	7.1 ± 1.7	6.9 ± 1.2	**0.001**
Day 7	6.3 ± 1.6	7.8 ± 1.7	6.4 ± 1.4	6.6 ± 1.9	0.592
1 month	6.9 ± 1.6	7.4 ± 2.4	6.8 ± 2.0	7.4 ± 1.8	0.628
**ESR (mm/hr)**					
Preoperative	17.6 ± 16.0	9.0 ± 8.5	11.4 ± 8.4	12.6 ± 7.9	0.083
Day 4	39.6 ± 20.3	20.5 ± 26.2	35.4 ± 18.9	38.4 ± 19.0	0.469
Day 7	42.1 ± 19.7	15.0 ± 18.4	37.7 ± 15.7	43.7 ± 15.8	0.113
1 month	26.8 ± 23.3	11.0 ± 12.7	22.7 ± 18.2	31.1 ± 23.6	0.355
**CRP (mg/L)**					
Preoperative	1.0 ± 1.1	0.3 ± 0.1	0.8 ± 1.1	0.8 ± 0.6	0.611
Day 4	46.9 ± 36.7	49.7 ± 66.0	49.5 ± 31.6	54.7 ± 43.1	0.882
Day 7	20.4 ± 18.5	9.8 ± 13.2	22.4 ± 15.6	31.4 ± 29.9	0.216
1 month	5.2 ± 13.2	1.1 ± 0.7	3.3 ± 4.2	8.2 ± 11.9	0.282
**Neutrophil (%)**					
Preoperative	55.8 ± 9.3	50.0 ± 10.5	58.0 ± 8.9	59.0 ± 8.0	0.324
Day 4	62.0 ± 9.5	84.1 ± 10.0	61.8 ± 9.4	61.7 ± 9.2	**0.014**
Day 7	57.6 ± 10.2	63.7 ± 5.5	59.0 ± 9.6	59.6 ± 10.1	0.774
1 month	54.9 ± 7.9	41.2 ± 30.1	56.8 ± 8.8	55.5 ± 4.2	0.074
**VAS back**					
Preoperative	6.5 ± 1.6	7.0 ± 1.4	6.4 ± 2.2	6.7 ± 1.5	0.893
1 Month	2.9 ±1.7	1.0 ± 1.4	2.9 ± 1.4	3.4 ± 1.9	0.199
1 Year	3.0 ± 1.8	1.0 ± 0.0	2.8 ± 1.7	3.6 ± 2.2	0.142
**VAS leg**					
Preoperative	6.9 ± 1.5	7.0 ± 1.4	7.0 ± 1.8	6.7 ± 1.4	0.914

1 Month	3.1 ± 1.9	1.0 ± 1.4	2.9 ± 1.5	3.3 ± 2.2	0.376
1 Year	3.0 ± 2.0	2.0 ± 2.8	2.9 ± 1.9	3.0 ± 2.4	0.928

Descriptive data represents mean ± standard deviation. Boldface indicates statistical significance. VB, vertebral body; WBC, white blood cell; ESR, erythrocyte sedimentation rate; CRP, C-reactive protein; VAS, visual analog scale.

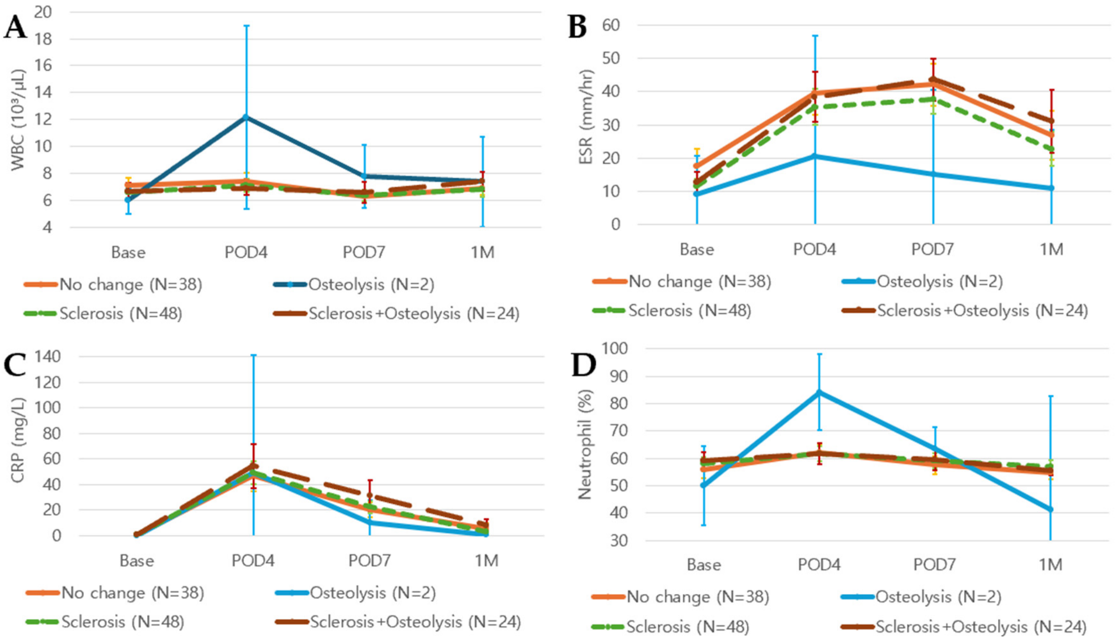

Supplementary Figure S1. Biochemical inflammatory marker trends over time according to adjacent vertebral body (VB) changes. We analyzed biochemical inflammatory markers dividing 4 groups according to VB changes (No change, n=38; Osteolysis, n=2; Sclerosis, n=48; Sclerosis+Osteolysis, n=24). WBC levels(10^3/μL) over time (A), ESR levels(mm/hr) over time (B), CRP levels(mg/L) over time (C), and neutrophil levels (%) over time (D). CRP, C-reactive protein; ESR, erythrocyte sedimentation rate; POD, postoperative day; WBC, white blood cell. Data are presented as mean values with 95% confidence interval.

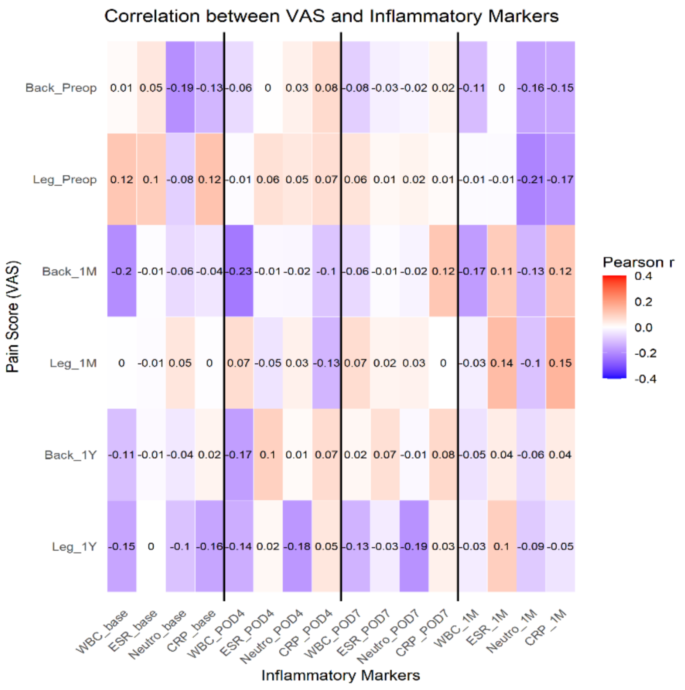

Supplementary Figure S2. Pearson correlation between inflammatory markers and pain scores. Pearson correlation analysis demonstrated no significant correlation between visual analog scale (VAS) scores (preoperative, 1 month postoperatively, 1 year postoperatively) and inflammatory markers (preoperative, 4 days postoperatively, 7 days postoperatively, 1 month postoperatively) (all |r| <.3).
